# Efficacy of integrated physical and psychological interventions on PTSD among forcibly displaced persons: a systematic review and meta-analysis

**DOI:** 10.1017/S0033291725000698

**Published:** 2025-04-07

**Authors:** Aditi Chaudhari, Apoorwa Chaudhari, Sandra O’Frans, Rohan Jayasuriya, Alvin Kuowei Tay

**Affiliations:** 1 School of Population Health, University of New South Wales, Sydney, Australia; 2 School of Psychology, University of New South Wales, Sydney, Australia; 3 Melbourne School of Population and Global Health, University of Melbourne, Melbourne, Australia; 4 Discipline of Psychiatry and Mental Health, School of Clinical Medicine, University of New South Wales, Sydney, Australia

**Keywords:** Forcibly displaced persons (FDPs), refugee, asylum seeker, internally discplaced persons, PTSD, integrated interventions, integrative, transdisciplinary, multidisciplinary

## Abstract

Forcibly displaced persons (FDPs) exposed to torture and trauma require multidisciplinary therapies to address their complex needs in mental and physical health. In this systematic review and meta-analysis, we explored the efficacy of models of care that integrated psychological and physical interventions for PTSD outcomes. We searched the Cochrane Central Register of Controlled Trials, Cochrane Database of Systematic Reviews, PubMed, EMBASE, CINAHL, PsychINFO, and Web of Science databases. We performed the meta-analysis on studies with randomized controlled trials and non-randomized controlled trial designs, followed by a subgroup analysis of moderators. In all meta-analyses, a random-effects model was used with standardized mean differences to accommodate for the heterogeneity of studies and outcome measures. In a meta-analysis of a between-group analysis of 11 studies comprising 610 participants, integrated intervention showed a moderate effect size (Hedges’ *g* = −0.46 (95% CI −0.80 to −0.12) in reducing PTSD symptoms. The proportion of variation in observed effects reflects 82% of variation in true effects (*I*^2^ = 82%). The efficacy of transdisciplinary interventions was higher compared to multidisciplinary models. Moderator analysis found that the type of PTSD measure, format of intervention, and type of personnel providing the intervention were significant predictors of efficacy. Integrated interventions are efficacious in reducing PTSD outcomes for people with FDPs and those exposed to war trauma. Factors such as the type of integration of interventions and service delivery need to be further studied with high-quality designs and larger numbers in future studies.

## Introduction

Humanitarian crises worldwide have led to the highest number of displaced populations ever recorded, with 110 million persons forcibly displaced in 2023 (UNCHR, 2023). This group, referred to as forcibly displaced persons (FDPs), includes refugees, asylum seekers, and internally displaced individuals driven from their homes due to persecution, conflict, violence, human rights violations, and public disorder (UNCHR, 2023). FDPs face high risks of complex psychiatric and physical comorbidities due to exposure to conflict and related stressors (Steel et al., 2009; Charlson et al., 2019; Miller & Rasmussen, 2017; Uphoff et al., 2020; Abu Suhaiban, Grasser, & Javanbakht, 2019; Silove, Ventevogel, & Rees, 2017; Rohlof, Knipscheer & Kleber, 2014). Despite the range of psychosomatic and mental disorders affecting conflict-affected populations, most studies have focused on PTSD, which is found to be 10 times more common in refugees and asylum seekers compared to host populations (Bogic, Njoku, & Priebe, 2015; Fazel, Wheeler, & Danesh, 2005). Some have found that the high prevalence of PTSD is especially notable among refugees who have faced severe trauma or torture (Abu Suhaiban et al., 2019).

Although refugees generally receive healthcare services in host countries, victims of torture or severe trauma require more specialized treatments to address their complex needs (Lambert & Alhassoon, 2015; Drožđek, Kamperman, Bolwerk, Tol, & Kleber, 2015; Abu Suhaiban et al., 2019). In many ethnic groups, such as those from South-East Asia, PTSD often manifests through somatization (e.g. physical complaints) rather than verbalized psychological distress (Hollifield, Warner, Lian, & Jenkins, 2002), necessitating a range of psychological, somatic, medical, and social interventions (Tay et al., 2019). Evidence suggests that utilizing an integrated approach, where affect can be processed through the body, as well as through a cognitive process, can increase efficacy of clinical interventions in the treatment of trauma (Ament-Lemke, 2018). However, in practice, many interventions have traditionally been siloed, either addressing physical conditions without acknowledging psychological distress or focusing on mental health without considering somatic symptoms. Given the interplay between psychological and physical symptoms and cultural influences on healthcare access, innovative, cross-disciplinary, integrated service models are needed to effectively address the health needs of FDPs (Kira & Tummala-Narra, 2014; Asfaw et al., 2020; White, Solid, Hodges, & Boehm, 2015; Abu Suhaiban et al., 2019; Rohlof, Knipscheer, & Kleber, 2014).

Empirical reviews and design of integrated care for FDPs are limited by variations in terms, frameworks, and approaches (Coulter, Khorsan, Crawford, & Hsiao, 2010). While there has been a growing use of cross-disciplinary collaborative care models, there is ambiguity in the taxonomy related to integrated care (Berman, Miller, Rosen, & Bicchieri, 2000; Choi & Pak, 2006; Sell, Hommes, Fischer, & Arnold, 2022). The National Institute of Mental Health (2021) defines integrated care as combining both primary health care and mental health care in one cohesive setting, and some authors have followed this definition (Abu Suhaiban et al., 2019; Daniel et al., 2023). Others, including Stein and Reider (2009) and Valentijn, Schepman, Opheij, and Bruijnzeels (2013), note that integration can take multiple forms, involving collaboration across professions, disciplines, and clinicians.

There is, however, reasonable consensus in the literature, particularly in the field of rehabilitation (York, Rainforth, & Giangreco, 1990; Körner, 2010; Nijhuis et al., 2007), in the distinction between multidisciplinary, interdisciplinary, and transdisciplinary models of collaboration across disciplines and roles (Berman et al., 2000; Körner, 2010; York et al., 1990). Current evidence from areas outside of mental health, indicate that transdisciplinary care is the most collaborative and efficacious in these settings. The authors of this review have drawn on reviews by Rosenfield (1992), Choi and Pak (2006), Khalili, Gilbert, Lising, MacMillan, and Xyrichis (2021), and Sell et al. (2022) to identify key indicators in classifying and delineating between multi-, inter-, and trans-disciplinary models (see Supplementary Material 1). In addition, different interventions have unique inherent characteristics, such as the type of personnel who deliver the intervention and the mode of delivery (e.g. group or individual), which may influence the outcome. Therefore, in assessing the efficacy of the intervention, a review needs to examine such moderating factors. To the best of our knowledge, the efficacy of these intervention models and effects of moderating factors has not been explored in the literature for the management of mental health of FDPs.

Previous systematic reviews of mental health interventions for FDPs have been limited to psychotherapeutic or psychosocial interventions only (Uphoff et al., 2020; Crumlish & O’Rourke, 2010; Lambert & Alhassoon, 2015; Palic & Elklit, 2011; Slobodin & de Jong, 2015; Turrini et al., 2019; Nosè et al., 2017; Thompson, Vidgen, & Roberts, 2018; Kip, Priebe, Holling, & Morina, 2020). Five meta-analyses have studied psychological interventions for PTSD in adult refugees (Kip et al., 2020; Lambert & Alhassoon, 2015; Nosè et al., 2017; Thompson et al., 2018; Turrini et al., 2019). Of these, only the review by Slobodin and de Jong (2014) reported on the efficacy of multidisciplinary interventions, which they defined as including psychotherapy, psychopharmacological treatment, and/or physiotherapy. While this study reported positive effects, the review included only three studies that involved any kind of somatic intervention (physiotherapy or non-verbal therapy). A review by Purgato et al. (2021) found that physical activity alone improves mental health outcomes in refugees but did not include integrated interventions and mostly involved migrants not exposed to prolonged trauma. Another systematic review by Thomas, Thirlaway, Bowes, and Meyers (2020) provided evidence that incorporating physical activity into psychotherapeutic approaches has a positive effect on psychological symptoms; however, none of the 22 RCT studies included in their review included refugee or war-affected populations.

This systematic review and meta-analysis aims to (i) assess the efficacy of integrated interventions on PTSD in FDPs (refugees, asylum seekers, internally displaced people, and non-displaced individuals exposed to conflict); (ii) evaluate different intervention models’ efficacy; and (iii) examine other factors that moderate intervention efficacy, particularly those related to service delivery.

## Methods

The protocol and reporting of this systematic review and meta-analysis followed the 2020 Preferred Reporting Items for Systematic Reviews and Meta-Analyses (PRISMA) guidelines. The Protocol was registered at the International Prospective Register of Systematic Reviews (CRD42022312905).

### Search strategy

We searched the Cochrane Central Register of Controlled Trials (CENTRAL), Cochrane Database of Systematic Reviews, PubMed, EMBASE, CINAHL, PsychINFO, and Web of Science data bases, for relevant articles from 2000 to 22 January 2022 and in English. Concepts for the search terms included (i) Population: refugee, asylum seeker, displaced populations, conflict-affected populations, (ii) Outcomes: mental or physical health and wellbeing outcomes, (iii) Interventions: integrated physical and psychological interventions. The search terms used are available in the Supplementary Material 3. We also conducted a manual forward and backward citation tracking of relevant papers to identify publications not covered by the original database searches. To ensure up-to-date search, we supplemented the search to December 2023 (see Supplementary Material 2).

**Figure 1.**
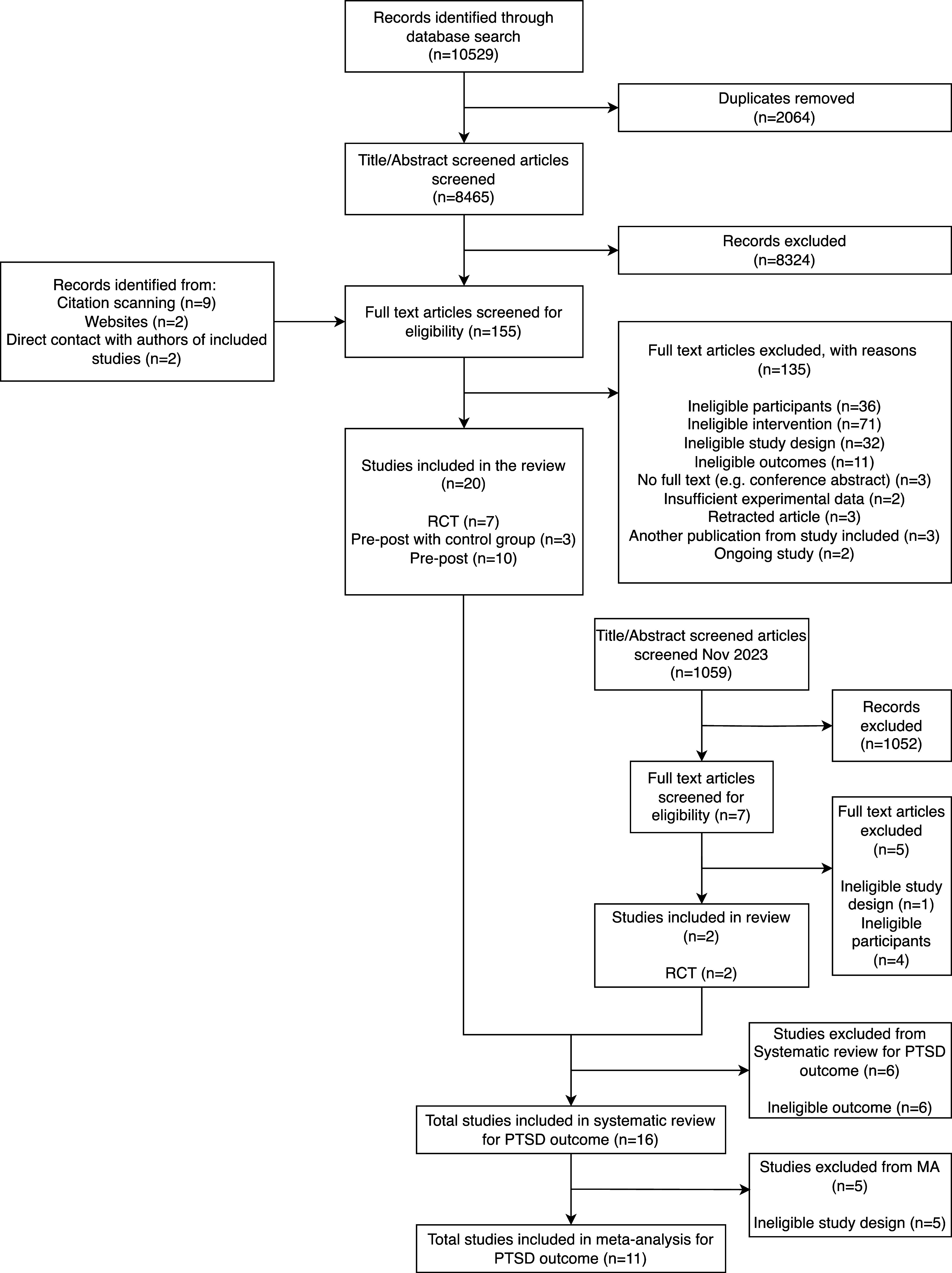
PRISMA diagram.

Citations and abstracts of all retrieved studies were imported into Covidence systematic review software (n.d) (Veritas Health Innovation, Melbourne, Australia). Three review authors (AC, APC, and SOF) independently screened for inclusion titles and abstracts of all records using preestablished criteria. Any disagreement on the inclusion of a study was arbitrated by a fourth author (RJ) and resolved by consensus. When titles and abstracts did not provide information on the inclusion and exclusion criteria, full articles were obtained to verify eligibility. Full-text articles were retrieved for all preliminarily selected studies. Full-text articles were inspected by the same three review authors for selection, and disputes were arbitrated by the same fourth author. We recorded the selection process, including reasons for exclusion, in sufficient detail to complete a PRISMA flow diagram (see Figure 1).

### Criteria for selection

See Supplementary Material 2 for the selection criteria. In our initial database search, we included studies with any mental or physical health and wellbeing outcomes; however, in this systematic review and meta-analysis, we presented only studies with PTSD outcomes based on the specific relevance of this outcome for the target population. Additionally this was the only outcome for which at least 10 studies provided data, which allows for appropriate investigation of heterogeneity in this data set with diverse variables (Myung, 2023; Deeks, Higgins, Altman, McKenzie, & Veroniki, 2024). To obtain high-quality studies for the meta-analysis, we opted to only include studies with RCT or non-RCT design. We contacted authors to obtain additional information where required. In addition, though proposed in the protocol, the inclusion of quasi-experimental pre-post studies was also omitted, as we were not able to attain the necessary unpublished data from many of the authors of the pre–post-studies.

### Data extraction

A comprehensive data extraction form was developed (AC) and refined (AC and RJ) based on the guidelines in the Cochrane Handbook for Systematic Reviews of Interventions and the TiDier framework (Hoffmann et al., 2014). The form was piloted on a subset of the included studies to ensure reliability and reproducibility, and then data from the included publications – e.g. participants, methods, intervention and control characteristics, outcome data – were extracted by three reviewers (APC, SOF, and RJ) independently and cross-checked for consistency by a third reviewer (AC). Where the means and standard deviation were not published (or provided to us by the authors), we used previously validated methods to extract means and SD; namely, data were computed from the P values or CIs provided in the study.

### Risk-of-bias assessment

Risk-of-bias assessment and quality of evidence was assessed using the Cochrane risk-of-bias version 2 tool (Higgins et al., 2011) for randomized trials and non-randomized trials with control groups. Two investigators (SOF and RJ) independently assessed each study as having low risk of bias, some concerns, or high risk of bias. Any discrepancies between the two evaluations were resolved by consensus after consulting a third investigator (AC). The criteria and results used for the risk-of-bias assessment are available in Supplementary Material 6.

### Data analysis

To examine the efficacy of integrated intervention in reducing mental distress, we performed meta-analyses for PTSD outcomes. Hedges’ *g* was calculated from the post-intervention or follow-up means, SDs, and sample sizes for each comparison of interest. Hedges’ *g* takes into account bias associated with small sample sizes (Hedges, Olkin, & Hedges, 1985). We analyzed the identified studies using the intention-to-treat principle, weighed the studies using the inverse-variance method, used a random-effects model to undertake meta-analytic pooling, and produced forest plots. Cochrane’s *Q* was used to test whether the effect sizes were heterogeneous between studies, and the *I*^2^ statistic was used to determine the percentage of variability attributable to between-study heterogeneity rather than sampling error. The *I*^2^ values of 25%, 50%, and 75% were taken to represent low, moderate, and high levels of heterogeneity, respectively (Higgins, Thompson, Deeks, & Altman, 2003).

Subgroup analyses were undertaken to assess the influence of potential moderators on overall effect size to explain the possible causes of heterogeneity. Potential moderators selected are presented in the results. Moderators were considered only if they were available in all independent studies. As the sample size of studies in the meta-analysis was small (n = 11), meta-regression was not undertaken.

Publication bias was assessed to test the assumption that studies with larger samples are more likely to be published. Both visual inspection of the funnel plots and statistical tests were used. Egger’s regression test (one-tailed p of <0.05 was considered to indicate the presence of the bias) (Egger, Davey Smith, Schneider, & Minder, 1997). We also used the trim-and-fill method from Duval and Tweedie (Peters, Sutton, Jones, Abrams, & Rushton, 2007) to determine the nature of potential publication bias and to compute an estimated effect size that accounts for it.

Meta-analyses, including subgroup analysis, was performed using STATA (version 18).

## Results

Database searches identified 8465 unique records following de-duplication in the initial search. After assessment of the titles and abstracts, a total of 142 full texts were screened. Thirteen additional studies were identified for full-text review through manual methods, such as screening of relevant papers and author publication lists. In total, 20 studies were eligible for inclusion to the review. The second database search in 2023 retrieved 1059 unique records, of which 7 full texts were reviewed and 2 included into the study. A total of 22 studies were included in the review. Of these, 16 studies were selected for our systematic review of PTSD outcomes, of which 11 were also included in our meta-analysis. A PRISMA flow diagram (Liberati et al., 2009) detailing the search process is presented in Figure 1.

### Study characteristics

A summary of the characteristics of the included studies for this review and meta-analysis is presented in Table 1.

**Table 1.** Study characteristics and intervention implementation

Authors *Country*	Study participants	Study design	Intervention	Outcomes assessed	Implementation		
					Dose	Format	Personnel
*Aizik-Reebs, Yuval, Hadash, Gebreyohans Gebremariam, and Bernstein (2021) *Israel*	Community sample of Eritrean asylum seekers residing in the Middle East (Israel). Over half (52.3%) participant with history of torture.	RCT Int = 98/78 Control = 60 (Waitlist)	Mindfulness-Based Trauma Recovery for Refugees (MBTR-R)	PTSD (HTQ) Depression symptoms (PHQ–9)	90 min sessions, twice weekly, × 6 weeks (12 sessions total)	Group	Mindfulness teachers with training in clinical or counselling psychology
Carlsson, Olsen, Kastrup, and Mortensen (2010) *Denmark*	Refugees or Asylum seekers with exposure to torture (or having a spouse exposed to torture)	Pre-post Int = 45	Multidisciplinary rehabilitation program	PTSD (HTQ) Anxiety (HSCL–25) Depression (HRSD) HRQOL (WHOQOL)	Varied - as required for individual	Individual	Local psychotherapist, physiotherapist, counsellors, and medical teams at RCT
*Drožđek, Kamperman, Bolwerk, Tol, and Kleber (2012) *Netherlands*	Male asylum seekers speaking Farsi/Dari with history of torture in their country of origin diagnosed with chronic PTSD according to DSM-IV	Pre-post with control group Int = 56 Control = 16 (Waitlist: medication only)	Den Bosch Group treatment	PTSD (HTQ) Anxiety and depression (HSCL–25)	2–3x weekly (frequency varied by type of group) × 52 weeks Psychotherapy sessions = 90 min Non-verbal therapy sessions = 75 min	Groups of 8–9 (same gender)	Group psychotherapy: led by two facilitators Nonverbal therapies: nonverbal therapist and a sociotherapists
Gordon, Staples, Blyta, and Bytyqi (2004) *Kosovo*	War-traumatized high school students in Kosovo	Pre-post Int = 136	Mind–Body Skills Group (MBSG)	PTSD (PTSD Reaction Index)	3 hr session, once weekly × 6 weeks	Groups of 41, 47 & 51	School teachers trained and supervised by CMBM’s psychiatrists and psychologists
*Gordon, Staples, Blyta, Bytyqi, and Wilson (2008) *Kosovo*	War-traumatized high school students in Kosovo screened using the 16 trauma symptom questions of the HTQ (measuring torture, trauma and DSM–5 PTSD symptoms)	RCT Int = 38 Control = 40 (Waitlist: delayed intervention)	Mind–Body Skills Group (MBSG)	PTSD (HTQ)	2 hr session twice-weekly × 6 weeks (12 sessions total)	Groups of 10	School teachers trained and supervised by CMBM’s psychiatrists and psychologists
Gordon, Staples, He, and Abdel Atti (2016) *Gaza*	Palestinian adults meeting the DSM–IV criteria for PTSD according to baseline symptom scores on the Harvard Trauma Questionnaire (HTQ)	Pre-post Int = 92	Mind–Body Skills Group (MBSG)	PTSD (HTQ) HSCL–25 (Anxiety) HSCL–25 (Depression) QOL (WHOQOL-BREF)	2 hr session weekly × 5 weeks (5 sessions total)	Groups of 5–11	Local teachers and clinicians were trained and supervised in delivering MBSGs and by CMBM physicians, social workers, psychologists, nurses, and educators
*Harlacher et al. (2019) *Cambodia*	Randomly chosen survivors of the Khmer Rouge regime diagnosed with PTSD according to ICD–10 research criteria and with pain-PTSD comorbidity	RCT Int = 55 Control = 58 (Waitlist)	Pain school	PTSD (PCL) Depression (HSCL–25)	2 hr session once weekly × 10 weeks (10 sessions total)	Groups of 10	Physiotherapists, physicians, social workers and psychologists at TPO trained to deliver the ‘Pain school’ program by DIGNITY psychiatrist, psychologist, physiotherapist, and project manager in a 1-week training workshop
*Hasha et al. (2020) *Norway*	Syrian refugees living in Bergen and adjacent municipalities, with pain symptoms and/or mental health problems	RCT Int = 27 Control = 23 (Waitlist: delayed intervention)	Physiotherapy Activity and Awareness Intervention (PAAI)	Subjective distress (IES-R 22) Common Mental Disorders (GHQ–12)	1 hr sessions weekly × 8 weeks (8 sessions total)	Groups of 10–12	Local physiotherapists
*Eskici, Hinton, Jalal, Yurtbakan, and Acarturk (2023) *Turkey*	Arabic speaking, Syrian women with a temporary protection status having psychological distress as indicated by a score of 1.75 or higher on the Hopkins Symptom Checklist–25	RCT Int =12 Control = 11 (TAU: psychological therapies only)	Culturally adapted cognitive-behavioral therapy (CA-CBT)	PTSD (HTQ) Anxious-depressive distress (HSCL–25)	2 hour sessions, once weekly × 7 weeks (7 sessions total)	Group of 12	Lay persons without formal mental health training were received 1-week training and weekly supervision by two experienced clinical psychologists
*Kananian et al. (2020) *Germany*	Afghan refugees from refugee camps with a diagnosis of trauma- and stressor-related disorder, depressive disorder, anxiety disorders, or somatoform disorder, per the criteria in the Diagnostic and Statistical Manual of Mental Disorders	RCT Int = 12/11 Control = 12 (Waitlist)	Culturally Adapted Cognitive Behavioral Therapy Plus Problem Management (CA-CBT+)	PTSD Symptoms (PCL–5) Depressive Symptoms (PHQ)	2.5-hr weekly sessions × 9 weeks (9 sessions total)	Group	Therapists with 2-day training in CA-CBT+
*Nordbrandt, Sonne, Mortensen, and Carlsson (2020) *Denmark*	Refugee or family reunified with a refugee; Having experienced a psychological trauma in the past (e.g., imprisonment, torture, political persecution, or war experiences) and diagnosed with PTSD according to ICD–10 research criteria	RCT (3-armed) Control =104) (TAU alone) IntB = 105 (TAU + basic body awareness therapy) IntM = 109 (TAU + mixed physical activity)	Multidisciplinary Program	PTSD (HTQ)* Depression (HSCL–25, HRSD)	Physiotherapy: 1 hr weekly × 20 weeks (20 sessions) Psychology: 16 total sessions Medical: 10 total sessions	Individual	General practitioner, psychologist, physiotherapist (only certified BBAT physiotherapists teaching BBAT)
Palic et al. (2009) *Denmark*	Refugees admitted for treatment at the CTR	Pre-post Int = 26	Multidisciplinary treatment	PTSD (HTQ)* Function (GAF)* Psychological distress (TSC–23) Anxiety (TSC–23) Depression (TSC–23) Somatization (TSC–23) Levels of social support (CSS)	Psychotherapy: 1x weekly for 16–18 weeks Physiotherapy: 1x weekly for 16–18 weeks	Individual	Psychologists and physiotherapists from CTR. Participants also were concurrently receiving care from municipality GP and social workers
*Shaw, Ward, Pillai, and Hinton (2019) *Malaysia*	Adult Dari-speaking female refugees from Afghanistan living in Kuala Lumpur symptomatic for emotional distress Refugee Health Screening score equal or greater to 12 on items 1–14)	RCT Int = 30 Control = 9 (Waitlist: delayed intervention)	Culturally adapted cognitive-behavioral therapy (CA-CBT)	Depression (HSCL–25) PTSD (HTQ)	1x weekly sessions for 8 weeks (8 sessions total)	Groups of 9–10	Lay persons from the community without formal mental health training
Stade et al. (2015) *Denmark*	Adult refugee (having come to Denmark as either an asylum seeker or as a quota refugee) or having been reunited with a refugee through family reunification and having trauma-related mental health problems	Pre-post Int = 9	Basic Body Awareness Therapy (BBAT)	Anxiety (HSCL)–25 Depression (HSCL)–25 PTSD (HTQ) Somatization (SCL–90) Function (SDS) QOL/Wellbeing (WHO–5) Quality of movement (BARS-MH) Pain (VAS)	1 × 90 min sessions weekly for 14 weeks (14 sessions total)	Groups (same gender)	Local physiotherapists trained in BBAT
*Tol et al. (2009) *Nepal*	All help-seeking clients at the Centre for Victims of Torture, Nepal (CVICT)	Pre-post with comparison group Int = 62 Control = 45 (Psychoeducation only)	Multidisciplinary program	PTSD Checklist-Civilian Version (PCL-C) Depression (BPRS)	Varied – as required for individual	Individual	Doctor, psychologist, psychiatrist, physiotherapist and counsellors. Counsellors included: one psychologist, two nurses and one yoga instructor trained in psychosocial counselling
*Wang et al. (2017) *Kosovo*	Adult traumatized victims of torture and war fulfilling DSM-IV criteria of comorbid chronic pain and one of the affective disorders: post-traumatic stress disorder (PTSD), depression or anxiety	RCT Int = 13 Control = 15 (Waitlist: delayed intervention)	Multidisciplinary program	PTSD (HTQ) Depression (HSCL–25)	BF-CBT sessions: 90 min (60-min CBT, 20-min biofeedback exercise) weekly sessions × 10 weeksPhysiotherapy: 60–90 min sessions weekly × 10 weeks	Individual BF-CBT, group physiotherapy	Two psychologists, three physiotherapists

#### Sample size and study design

Of the 16 studies, 14 were RCTs, and 2 were non-randomized control trials (Table 1). One thousand two hundred and ninety-seven participants were included in the review, of which 904 received intervention, and 393 were part of control or comparison groups (Table 1). Eight studies had small sample sizes (n<50) and the sample sizes ranged from 318 to 9 participants (Table 1).

#### Participants

Study populations included refugee and/or asylum seekers (10 studies), populations with exposure to conflict-related trauma without displacement (7 studies), and torture survivors (4 studies) (Table 1). Participants included those with a formal diagnosis of PTSD and/or depression (11 studies) and those without (4 studies) (Table 1).

#### Setting

Studies were from 11 different countries. Countries from Europe contributed most (n = 10) studies and Asia contributed the remaining six. Most studies (n = 13) were conducted in high- to upper–middle-income countries and three were from lower- to lower–middle-income countries, according to the World Bank classification (The World Bank, 2022). Interventions were conducted across a range of settings, including outpatient clinics, community groups, and schools.

### Intervention characteristics

There was considerable inter-study variation in the integration level, content, and dose of therapy provided across intervention designs.

Frequency of sessions ranged from 1 to 5 days per week and total intervention duration ranged between 6 and 52 weeks. Two studies (Tol et al., 2009; Carlsson et al., 2005) provided therapy to participants ‘as required’ based on participants’ individual needs. Interventions were delivered individually (n = 4), in groups (n = 11) or as a combination of both (n = 1) (Table 1). Interventions were delivered either by health professionals (n = 12), paraprofessionals, including local teachers or community members with no previous professional education, or as a combination of both (n = 2) (Table 1).

The interventions identified were delivered in various cross-disciplinary forms (e.g. multidisciplinary, interdisciplinary, and transdisciplinary). See Supplementary Material 1 for definitions of the various categories and the table of indicators that we used to classify the interventions into these groups. In this review, we identified 5 multidisciplinary, 1 interdisciplinary, and 9 transdisciplinary interventions. Of the transdisciplinary interventions, 4 of 9 studies were stand-alone mindfulness-based programs aligning with Kabat-Zinn’s (1982) model of mindfulness-based stress reduction, and three others looked at culturally adapted cognitive behavior therapy program based on Hinton et al.’s (2005) framework. The remaining 2 transdisciplinary programs were physiotherapy-based programs with Palic and Elklit (2009) implementing ‘Basic Body Awareness Therapy’, which is a movement awareness training program and Hasha et al. (2020)’s PAAI program, which integrates conventional physiotherapy exercises with mindfulness, relaxation, and breathing techniques exercises. There was one interdisciplinary study, which included only education as the only modality of intervention.

Psychotherapy, counselling, psychoeducation, and cognitive behavioral therapy were commonly reported psychological interventions; physiotherapy was the most commonly reported somatic intervention; and concurrent pharmacological therapy was reported in at least some participants in four studies (see Supplementary Material 5).

More information on the intervention characteristics of the included studies can be found in Table 1 and in Supplementary Material 5.

### Risk of bias

The Cochrane ROB-2 tool determined that there was high risk of bias in 2 studies, some risk in 6 studies, and low risk in 3 studies. See Supplementary Material 6 for Risk of Bias table.

### Meta analysis

**Figure 2.**
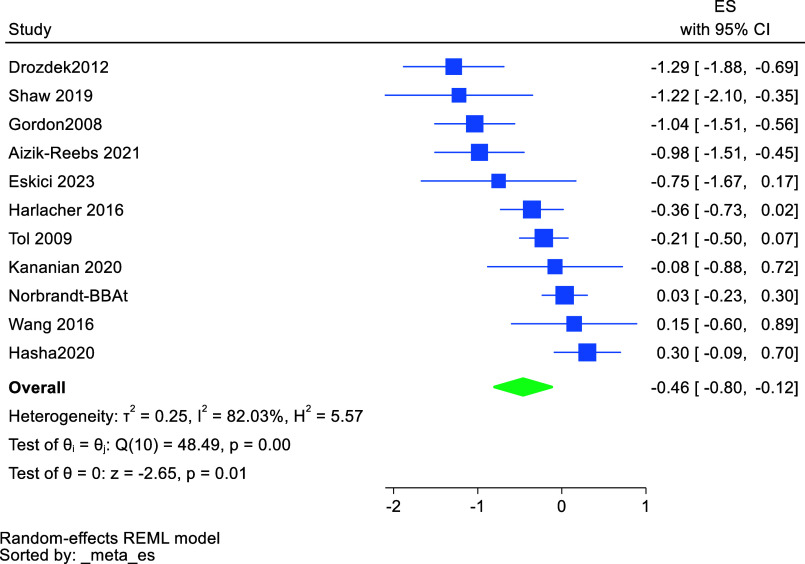
Forest plot of distribution of effect sizes for PTSD after integrated interventions.

This meta-analysis was based on 11 studies and 610 participants. The effect sizes and their confidence intervals and heterogeneity of studies included in the meta-analysis are presented in a forest plot for PTSD in Figure 2. The between-group analysis comparing the integrated intervention group and control group, using random-effects analyses yielded an overall effect size of Hedges’ *g* = −0.46 (95% CI −0.80 to −0.12); P = <0.01. This indicates a significant positive treatment effect resulting in reduction of PTSD in the group given the integrated intervention. As recommended (Morris, 2023), the results of several tests for heterogeneity are presented in Figure 2. The *Q* test for heterogeneity revealed a chi-square value of 48.5 (df = 10; P < 0.001), indicating the presence of heterogeneity. The I squared percentage (*I*^2^) suggest that 82% of variance in treatment effect was due to heterogeneity and only 18% due to chance (sampling effects), when the full sample is considered. This high level of heterogeneity was explored in our subgroup analysis of potentially moderating factors.

### Subgroup analysis

Based on prior literature and description of included interventions, potential moderators of the effect size were identified. The results are presented in Table 2, and forest plots of the subgroup analysis are presented in Supplementary Material 8. The subgroup analysis found that certain factors, such as the PTSD measure (HTQ), group delivery format, and delivery by paraprofessionals, significantly influenced treatment effects, while other factors like study design, integration type, and intervention volume showed no significant differences.

**Table 2.** Subgroup analysis

Moderator	Subgroup	k	Hedges g	95% CI	%I^2^	Q-diff	P value
1. Control						0.55	0.76
	RCT: TAU	2	−0.243	−0.972	63.5		
				0.486			
	RCT: Waitlist	7	−0.449	−0.903	80.0		
				0.004			
	NRCT	2	−0.719	−1.775	90.4		
				0.338			
2. PTSD measure						4.71	0.03
	HTQ	7	−0.707	**−1.161**	78.8		
				**−0.254**			
	Other (PCL, IESR)	4	−0.099	−0.409	55.7		
				0.210			
3. Integration						5.48	0.06
	Multidisciplinary	3	−0.064	−0.272	9.0		
				0.143			
	Interdisciplinary	2	−0.796	−1.706	85.4		
				0.114			
	Transdisciplinary	6	−0.603	**−1.122**	77.9		
				**−0.084**			
4. Delivery: Group vs Individual						7.44	0.006
	Group*	9	−0.590	**−0.966**	80.2		
				**−0.213**			
	Individual	2	0.045	−0.212	0.00		
				0.302			
5.Delivery: Personnel						7.53	0.006
	Health professional	9	−0.33	−0.68	80.6		
				0.02			
	Other (non-health professional)	2	−1.08	**−1.49**	0.00		
				**−0.67**			
6.Delivery: Intensity/volume						0.39	0.53
	Moderate	7	−0.557	**−0.992**	77.2		
				**−0.122**			
	High	4	−0.319	−0.923	88.5		
				0.286			

Studies included in meta-analysis

*Factors related to the design of studies:*

(a) Study design (e.g. RCT TAU versus RCT Waitlist versus non-randomized controlled trials [NRCT]).

Effect sizes of all 3 subgroups were not significant, and subgroups were not different (Q-diff = 0.55; P = 0.76).

(b) Measure of PTSD (HTQ versus Others).

When subgroup analysis by type of PTSD measure was conducted, the subgroup of studies that used the HTQ (7 studies) yielded an overall effect size of Hedges’ *g* = −0.7 (95% CI −1.16 to −0.25), which was significant. In contrast, studies using other measures (e.g. PCL-5), yielded an overall effect size of Hedges’ *g* = −0.10 (95% CI −0.41 to 0.21). The effect sizes of subgroups were different (Q-diff = 4.71; P = 0.03).

(c) Types of Integration.

Among the three subgroups for type of integration, only the effect size for the transdisciplinary group (Hedges’ *g* = −0.6 [95% CI −1.12 to −0.084]) was significant. The effect sizes of subgroups were not different (Q-diff = 5.48; P = 0.06). The *I*^2^ percent indicated that the multidisciplinary group had much lower heterogeneity (*I*^2^ = 9%), compared to the other two groups, which had higher heterogeneity (*I*^2^ = 85% and 78%).

*Factors related to the delivery of the intervention:*

(d) Delivery format (Group versus Individual).

When the delivery was in a group setting, the effect size was significant (Hedges’ *g* = −0.59 [95% CI −0.97 to −0.21]). When delivered in an individual setting the effect size was not significant. A significant difference was seen between the groups (Q-diff = 7.44; P = 0.006).

(e) Delivery personnel (Health Professional versus Other).

When the intervention was delivered by other professionals (e.g. paraprofessional) the effect size was significant (Hedges’ *g* = −1.08 (95% CI −1.49 to −0.67). When delivered by health professionals the effect size was not significant. A significant difference was seen between the groups (Q-diff = 7.53; P = 0.006). There was wide difference in the *I*^2^ percent, with high heterogeneity in the group that had more studies in both cases. Additional evidence for the moderator effect of type of delivery (group versus individual) and delivery personnel (other personnel versus health professionals) is found as there is no overlap of the confidence intervals between the groups (Morris, 2023).

(f) Volume of intervention.

A measure for the intensity of intervention was created by considering the average duration of session and length of intervention period. Of the two subgroups, those who received a moderate intensity (600–1200 units) had a significant effect (Hedges’ *g* = −0.56 [95% CI −1.0 to −0.12]) but not the other group. The effect sizes of subgroups were not different (Q-diff = 0.39; P = 0.53).

### Publication bias

Publication bias was tested using three methods: (i) funnel plots (ii) trim and fill method, and (iii) Egger’s regression test. See Supplementary Material 7 for funnel plot and trim and fill figures. The funnel plot indicates that small studies were dispersed in both significant and non-significant areas, suggesting an absence of publication bias from small studies. This was further confirmed by the trim and fill analysis (Figure 7.1 in Supplementary Material 7), where the imputed study resulted in only a marginal reduction in effect size (from −0.46 to −0.38). In addition, Egger’s regression test for small-study effects was not significant (P = 0.22). Taken together, these results provide no evidence of publication bias (of small studies) in the meta-analysis.

## Discussion

To our knowledge, this is the first systematic inquiry into the effects of combined physical and psychological health interventions on PTSD among refugees, asylum seekers, internally displaced persons, and conflict-exposed populations, including survivors of torture and severe trauma. Our meta-analyses revealed that integrated interventions significantly reduced PTSD symptoms, although individual study outcomes exhibited considerable variability, with several studies presenting less conclusive results.

Our results are consistent with earlier research by Thomas et al. (2020), who have shown comparable findings for integrated interventions in non-FDP populations, and Slobodin and de Jong (2015), who reported the efficacy of multidisciplinary studies for FDPs from a much smaller sample of studies.

The strengths of our study include a comprehensive search for all relevant studies of heterogenous forms of physical and mental health interventions that involved FDPs. To overcome the limitation of previous meta-analyses by participant group (e.g. refugees in high-income countries: Nose et al., 2017), we included populations currently living as refugees, asylum seekers, and those displaced within their countries of origin. Furthermore, our study defined and categorized the different types of integration between psychological and physical interventions, and it is unique in that we included integrated models of care that combined psychological, physical and, in some cases, social components.

The secondary aim of this study was to compare alternative models of integration. Although literature suggests that interdisciplinary model enhance teamwork compared to models (Körner, 2010), and that transdisciplinary models better meet client needs compared to interdisciplinary models (Berman et al., 2000), no study has concurrently compared all three models. Our subgroup analysis showed that the transdisciplinary models had the highest effect size, while multidisciplinary models showed low, non-significant effects. Differences among the models was not significant, likely due to small sample sizes and minimal effect size differences.

There are known barriers to integration in a multidisciplinary model, due to professionals defending their disciplinary boundaries preventing knowledge sharing and impeding coordinated work (Atwal & Caldwell, 2002, Oborn & Dawson, 2010; Ferlie, Fitzgerald, Wood & Hawkins, 2005; Mackintosh & Sandall, 2010, Waring, Marshall, & Bishop, 2015). By contrast, these boundaries are eliminated in transdisciplinary work whereby each team member contributes and works toward shared goals and a single treatment plan (Perkins & Schensul, 2017; York et al., 1990; Berman et al., 2000; Körner, 2010).

Another key advantage in the case of transdisciplinary care is that the intervention can be implemented by task shifting and training, further reducing the abovementioned barriers or potential for conflicts about role overlaps and ownership of information between professionals from various disciplines. This is reflected in our findings, where the interventions described in transdisciplinary studies were delivered either by a single implementor or a group of implementors delivering the same intervention. In the group of multidisciplinary and interdisciplinary studies, interventions from various disciplines were delivered by different professionals from corresponding disciplines. However, these findings need to be interpreted with caution due to the small sample sizes of groups in the meta-analysis. There is a need for larger meta-analyses of high-quality studies that compare the different models of integration in delivering integrated solutions for this target group.

Our meta-analysis highlighted notable diversity in the implementation of integrated interventions, including outcome measurement, delivery format, and provider type. The moderator analysis revealed that group delivery formats were more effective than individual formats. Group settings likely provide additional information and motivation from peers (Ley, Rato Barrio, & Koch, 2018; Stade, Skammeritz, Hjortkjær, & Carlsson, 2015). Qualitative studies have reported that group formats offered social interaction, a platform for social support, and a sense of community belonging (Hasha et al., 2020; Ley et al., 2018; Piwowarczyk & Ona, 2019; Stade et al., 2015; Verreault, 2017). Rosendahl, Alldredge, Burlingame, and Strauss (2021)) found group therapy as effective as individual therapy across various symptoms and conditions, including PTSD.

We also found a significant difference in the efficacy of interventions provided by non-health professionals, such as paraprofessionals and lay workers, compared to health professionals. One possible reason could be that transdisciplinary interventions, often delivered by paraprofessionals, were more effective than multidisciplinary interventions provided by health professionals. In addition, interventions by peers or community members may increase trust, comfort, and reduce stigma (Palic & Elklit, 2009). Katigbak, Van Devanter, Islam, and Trinh-Shevrin (2015) recommended employing local health workers in mental health organizations to help patients overcome distrust and share similar life experiences with clients.

Several caveats associated with this review warrant further consideration. First, we included only English-language studies from 2000 to 2023, potentially excluding relevant research from other languages and timeframes. Due to the emerging nature of integrated interventions for this demographic, the number of eligible studies was small, limiting the power of moderator analyses. While all attempts were made to contact the authors of studies using pre–post designs, we had to exclude them from the analysis for several reasons. Some did not provide sufficient details of interventions; there were age group restrictions; many authors did not provide the raw data to compute effect size for within-group analyses.

As with all meta-analyses, our findings are constrained by the limitations of the included studies. Three studies had sample sizes below 30, and reporting on participant and trial characteristics was often insufficient. Key details, such as assessor blinding, co-interventions (e.g. pharmacotherapy), and treatment deviations, were frequently unclear, complicating the attribution of outcomes to the therapeutic intervention. In addition, inconsistent reporting on missing data handling limited the generalizability of findings.

Another limitation that contributed to heterogeneity among included studies included differences in country of origin, time since migration, trauma exposure, living conditions, and cultural background. Inconsistent reporting of these variables prevented using them in moderator analyses.

Inconsistent reporting precluded subgroup analyses on cultural adaptation, despite evidence that culturally adapted interventions may yield larger effect sizes (Harper Shehadeh, Heim, Chowdhary, Maercker, & Albanese, 2016). In addition, although conflict-affected populations experience diverse mental health issues, most studies focused on PTSD, limiting the scope of our findings (Turrini et al., 2021; Morina, Akhtar, Barth, & Schnyder, 2018).

Due to ethical and logistical challenges of implementing RCT in refugee populations, many studies have been conducted within assistance programs without randomization (Silove, Manicavasagar, Coello,& Aroche, 2005; McFarlane & Kaplan, 2012; Van Wyk & Schweitzer, 2013). Consequently, our meta-analysis included non-randomized controlled trials, increasing the risk of bias. Despite these limitations, this meta-analysis contributes significantly to the evidence base for integrated interventions. However, the mechanisms underlying their effectiveness remain unclear.

### Implications for policy and practice

Our findings underscore the need for a paradigm shift in healthcare policy for forcibly displaced trauma survivors, advocating for an integrated model that bridges the gap between physical and mental health services. This requires interdisciplinary collaboration, standardized training, and funding mechanisms that support holistic care. Policymakers should prioritize scalable, evidence-based interventions that leverage group-based and non-specialist delivery models, particularly in low-resource settings, where sustainability is a key challenge.

In practice, these insights call for adaptable, culturally responsive care programs that equip healthcare providers to address the complex needs of displaced populations. Integration efforts should foster collaboration among psychologists, physiotherapists, psychiatrists, and primary care providers to ensure a seamless continuum of care.

### Implications for research

The field urgently requires rigorous randomized trials to strengthen the evidence base for integrated interventions. While ethical and logistical challenges have often limited randomization in refugee studies, innovative trial designs – such as patient-centered randomization approaches – can enhance feasibility (Jadad & Rennie, 1998). Future research should also extend beyond PTSD-focused outcomes to capture the broader experiences of forced migration, incorporating culturally relevant measures of well-being and functioning. In addition, studies should explore mediators of treatment efficacy, cultural adaptations, and implementation strategies, with a particular emphasis on low- and middle-income countries, where critical knowledge gaps remain. By addressing these gaps, our research contributes to shaping policies and practices that support the long-term recovery and resilience of displaced trauma survivors.

## Conclusion

Integrated interventions with psychological and physical components are efficacious in reducing PTSD symptoms in forcibly displaced and war-trauma-affected people persons. Factors such as type of integration of interventions and service delivery need to be further studied with high-quality designs and larger numbers in future studies.

## Supporting information

Chaudhari et al. supplementary material Chaudhari et al. supplementary material

## Supplementary material

The supplementary material for this article can be found at http://doi.org/10.1017/S0033291725000698.

## References

[r1] Abu Suhaiban, H., Grasser, L. R., & Javanbakht, A. (2019). Mental health of refugees and torture survivors: A critical review of prevalence, predictors, and integrated care. International Journal of Environmental Research and Public Health, 16(13), 2309. doi:10.3390/ijerph16132309 31261840 PMC6651013

[r2] Aizik-Reebs, A., Yuval, K., Hadash, Y., Gebreyohans Gebremariam, S., & Bernstein, A. (2021). Mindfulness-based trauma recovery for refugees (MBTR-R): Randomized Waitlist-control evidence of efficacy and safety. Clinical Psychological Science, 9(6), 1164–1184. doi:10.1177/2167702621998641

[r3] Ament-Lemke, A. (2018). *Healing the mind and body: Practitioner perspectives on integrating cognitive and somatic approaches in psychotherapy with refugees, asylees and asylum seekers* . Social Work Master’s Clinical Research Paper, No. 813. University of St. Thomas. https://ir.stthomas.edu/ssw_mstrp/813

[r4] Asfaw, B. B., Beiersmann, C., Keck, V., Nikendei, C., Benson-Martin, J., Schütt, I., & Lohmann, J. (2020). Experiences of psychotherapists working with refugees in Germany: A qualitative study. BMC Psychiatry, 20 *(*1), 588. doi:10.1186/s12888-020-02996-0 33308187 PMC7733283

[r5] Atwal, A., & Caldwell, K. (2002). Do multidisciplinary integrated care pathways improve interprofessional collaboration?. Scandinavian Journal of Caring Sciences, 16(4), 360–367. doi:10.1046/j.1471-6712.2002.00101.x 12445105

[r6] Berman, S., Miller, A. C., Rosen, C., & Bicchieri, S. (2000). Assessment training and team functioning for treating children with disabilities. Archives of Physical Medicine and Rehabilitation, 81(5), 628–633. doi:10.1016/s0003-9993(00)90047-9 10807104

[r7] Bogic, M., Njoku, A., & Priebe, S. (2015). Long-term mental health of war-refugees: A systematic literature review. BMC International Health and Human Rights, 15, 29. doi:10.1186/s12914-015-0064-9 26510473 PMC4624599

[r8] Carlsson, J. M., Olsen, D. R., Kastrup, M. & Mortensen, E. L. (2010). Late mental health changes in tortured refugees in multidisciplinary treatment. The Journal of Nervous and Mental Disease, 198(11), 824–828. doi: 10.1097/NMD.0b013e3181f97be3. 21048474

[r9] Charlson, F., van Ommeren, M., Flaxman, A., Cornett, J., Whiteford, H., & Saxena, S. (2019). New WHO prevalence estimates of mental disorders in conflict settings: A systematic review and meta-analysis. Lancet (London, England), 394(10194), 240–248. doi:10.1016/S0140-6736(19)30934-1 31200992 PMC6657025

[r10] Covidence Systematic Review Software (n.d.). Veritas Health Innovation, Melbourne, Australia. www.covidence.org.

[r11] Choi, B. C., & Pak, A. W. (2006). Multidisciplinarity, interdisciplinarity and transdisciplinarity in health research, services, education and policy: 1. Definitions, objectives, and evidence of effectiveness. Clinical and Investigative Medicine. Medecine Clinique et Experimentale, 29(6), 351–364. 17330451

[r12] Coulter, I. D., Khorsan, R., Crawford, C., & Hsiao, A. F. (2010). Integrative health care under review: An emerging field. Journal of Manipulative and Physiological Therapeutics, 33(9), 690–710. 10.1016/j.jmpt.2010.08.007 21109060

[r13] Crumlish, N., & O’Rourke, K. (2010). A systematic review of treatments for post-traumatic stress disorder among refugees and asylum-seekers. The Journal of Nervous and Mental Disease, 198(4), 237–251. doi:10.1097/NMD.0b013e3181d61258 20386252

[r14] Daniel, K. E., Blackstone, S. R., Tan, J. S., Merkel, R. L., Hauck, F. R., & Allen, C. W. (2023). Integrated model of primary and mental healthcare for the refugee population served by an academic medical centre. Family Medicine and Community Health, 11(2), e002038. doi:10.1136/fmch-2022-002038 37012045 PMC10083854

[r15] Deeks, J. J., Higgins, J. P. T., Altman, D. G., McKenzie, J. E., & Veroniki, A. A. (Eds.). (2024). Chapter 10: Analysing data and undertaking meta-analyses. In Cochrane handbook for systematic reviews of interventions (version 6.3). Cochrane. https://training.cochrane.org/handbook/current/chapter-10

[r17] Drožđek, B., Kamperman, A. M., Bolwerk, N., Tol, W. A., & Kleber, R. J. (2012). Group therapy with male asylum seekers and refugees with posttraumatic stress disorder: A controlled comparison cohort study of three day-treatment programs. The Journal of Nervous and Mental Disease, 200(9), 758–765. doi:10.1097/NMD.0b013e318266f860 22922235

[r18] Egger, M., Davey Smith, G., Schneider, M., & Minder, C. (1997). Bias in meta-analysis detected by a simple, graphical test. BMJ (Clinical Research Ed.), 315(7109), 629–634. doi:10.1136/bmj.315.7109.629 PMC2127453 9310563

[r19] Eskici, H. S., Hinton, D. E., Jalal, B., Yurtbakan, T., & Acarturk, C. (2023). Culturally adapted cognitive behavioral therapy for Syrian refugee women in Turkey: A randomized controlled trial. Psychological Trauma: Theory, Research, Practice and Policy, 15(2), 189–198. doi:10.1037/tra0001138 34618479

[r20] Fazel, M., Wheeler, J., & Danesh, J. (2005). Prevalence of serious mental disorder in 7000 refugees resettled in western countries: A systematic review. Lancet (London, England), 365(9467), 1309–1314. doi:10.1016/S0140-6736(05)61027-6 15823380

[r21] Ferlie, E., Fitzgerald, L., Wood, M., & Hawkins, C. (2005). The nonspread of innovations: The mediating role of professionals. Academy of Management Journal, 48. doi:10.5465/AMJ.2005.15993150.

[r22] Gordon, J. S., Staples, J. K., Blyta, A., & Bytyqi, M. (2004). Treatment of posttraumatic stress disorder in postwar Kosovo high school students using mind-body skills groups: A pilot study. Journal of Traumatic Stress, 17(2), 143–147. 15141787 10.1023/B:JOTS.0000022620.13209.a0

[r23] Gordon, J. S., Staples, J. K., Blyta, A., Bytyqi, M., & Wilson, A. T. (2008). Treatment of posttraumatic stress disorder in postwar Kosovar adolescents using mind-body skills groups: A randomized controlled trial. The Journal of Clinical Psychiatry, 69(9), 1469–1476. doi:10.4088/JCP.v69n0915 18945398

[r24] Gordon, J. S., Staples, J. K., He, D. Y., & Abdel Atti, J. A. (2016) Mind-body skills groups for posttraumatic stress disorder in Palestinian adults in Gaza. Traumatology, 22(3) 155–164. doi:10.1037/trm0000081

[r25] Harlacher, U., Polatin, P., Taing, S., Phana, P., Sok, P., & Sothera, C. (2019). Education as treatment for chronic pain in survivors of trauma in cambodia: results of a randomized controlled outcome trial. International Journal of Conflict and Violence, 13, a655. doi:10.4119/ijcv-3124

[r26] Harper Shehadeh, M., Heim, E., Chowdhary, N., Maercker, A., & Albanese, E. (2016). Cultural adaptation of minimally guided interventions for common mental disorders: A systematic review and meta-analysis. JMIR Mental Health, 3(3), e44. 10.2196/mental.5776 27670598 PMC5057065

[r27] Hasha, W., Igland, J., Fadnes, L. T., Kumar, B., Haj-Younes, J., Strømme, E. M., Norstein, E. Z., Vårdal, R., & Diaz, E. (2020). The effect of physiotherapy group intervention in reducing pain disorders and mental health symptoms among syrian refugees: A randomized controlled trial. International Journal of Environmental Research and Public Health, 17(24), 9468. doi:10.3390/ijerph17249468 33348794 PMC7767069

[r28] Hedges, L. V., Olkin, I., & Hedges, L. V. (Eds.) (1985). Statistical methods for meta-analysis. Academic Press.

[r29] Higgins, J. P., Altman, D. G., Gøtzsche, P. C., Jüni, P., Moher, D., Oxman, A. D., Savovic, J., Schulz, K. F., Weeks, L., Sterne, J. A., Cochrane Bias Methods Group, & Cochrane Statistical Methods Group (2011). The cochrane collaboration’s tool for assessing risk of bias in randomised trials. BMJ (Clinical Research ed.), 343, d5928. doi:10.1136/bmj.d5928 PMC3196245 22008217

[r30] Higgins, J. P., Thompson, S. G., Deeks, J. J., & Altman, D. G. (2003). Measuring inconsistency in meta-analyses. BMJ (Clinical Research ed.), 327(7414), 557–560. doi:10.1136/bmj.327.7414.557 PMC192859 12958120

[r31] Hinton, D. E., Chhean, D., Pich, V., Safren, S. A., Hofmann, S. G., & Pollack, M. H. (2005). A randomized controlled trial of cognitive-behavior therapy for Cambodian refugees with treatment-resistant PTSD and panic attacks: A cross-over design. Journal of Traumatic Stress, 18(6), 617–629. doi:10.1002/jts.20070 16382423

[r32] Hoffmann, T. C., Glasziou, P. P., Boutron, I., Milne, R., Perera, R., Moher, D., … Michie, S. (2014). Better reporting of interventions. BMJ (Online), 348(3), g1687–g1687. doi:10.1136/bmj.g1687 24609605

[r33] Hollifield, M., Warner, T. D., Lian, N., & Jenkins, J. H. (2002). PTSD in refugees and asylum seekers: The role of somatic and cultural expression. Journal of Traumatic Stress, 15(4), 213–220. 10.1023/A:101601372284912092913

[r34] Jadad, A. R., & Rennie, D. (1998). The randomized controlled trial gets a middle-aged checkup. JAMA, 279(4), 319–320. 10.1001/jama.279.4.319 9450719

[r35] Kabat-Zinn, J. (1982). An outpatient program in behavioral medicine for chronic pain patients based on the practice of mindfulness meditation: theoretical considerations and preliminary results. General Hospital Psychiatry, 4(1), 33–47. doi:10.1016/0163-8343(82)90026-3 7042457

[r36] Kananian, S., Soltani, Y., Hinton, D., & Stangier, U. (2020). Culturally adapted cognitive behavioral therapy plus problem management (CA-CBT+) with afghan refugees: A randomized controlled Pilot study. Journal of Traumatic Stress, 33(6), 928–938. doi:10.1002/jts.22615 33155348

[r37] Katigbak, C., Van Devanter, N., Islam, N., & Trinh-Shevrin, C. (2015). Partners in health: A conceptual framework for the role of community health workers in facilitating patients’ adoption of healthy behaviors. American Journal of Public Health, 105(5), 872–880. doi:10.2105/AJPH.2014.302411 25790405 PMC4386525

[r38] Khalili, H., Gilbert, J., Lising, D., MacMillan, K. M., Xyrichis, A. (2021). Proposed lexicon for the interprofessional field. A reprint publication by Interprofessional Research Global. https://interprofessionalresearch.global/

[r39] Kip, A., Priebe, S., Holling, H., & Morina, N. (2020). Psychological interventions for posttraumatic stress disorder and depression in refugees: A meta-analysis of randomized controlled trials. Clinical Psychology & Psychotherapy, 27(4), 489–503. doi:10.1002/cpp.2446 32191370

[r40] Kira, I., & Tummala-Narra, P. (2014). Psychotherapy with refugees: Emerging paradigms. Journal of Loss and Trauma: International Perspectives on Stress & Coping., Online First Publication.

[r41] Körner, M. (2010). Interprofessional teamwork in medical rehabilitation: A comparison of multidisciplinary and interdisciplinary team approach. Clinical Rehabilitation, 24(8), 745–755. doi:10.1177/0269215510367538 20530646

[r42] Lambert, J. E., & Alhassoon, O. M. (2015). Trauma-focused therapy for refugees: Meta-analytic findings. Journal of Counselling Psychology, 62(1), 28–37. doi:10.1037/cou0000048 25485547

[r43] Ley, C., Rato Barrio, M., & Koch, A. (2018). “In the Sport I Am Here”: Therapeutic processes and health effects of sport and exercise on PTSD. Qualitative Health Research, 28(3), 491–507. doi:10.1177/1049732317744533 29199529 PMC5764144

[r44] Liberati, A., Altman, D. G., Tetzlaff, J., Mulrow, C., Gotzsche, P. C., Ioannidis, J. P. A., … Moher, D. (2009). The PRISMA statement for reporting systematic reviews and meta-analyses of studies that evaluate healthcare interventions: Explanation and elaboration. BMJ (Online), 339. doi:10.1136/bmj.b2700 PMC2714672 19622552

[r45] Mackintosh, N., & Sandall, J. (2010). Overcoming gendered and professional hierarchies in order to facilitate escalation of care in emergency situations: The role of standardised communication protocols. Social Science & Medicine, 71(9), 1683–1686. doi:10.1016/j.socscimed.2010.07.037 20850919

[r46] McFarlane, C. A., & Kaplan, I. (2012). Evidence-based psychological interventions for adult survivors of torture and trauma: A 30-year review. Transcultural Psychiatry, 49, 539–567. 23008355 10.1177/1363461512447608

[r47] Miller, K. E., & Rasmussen, A. (2017). The mental health of civilians displaced by armed conflict: An ecological model of refugee distress. Epidemiology and Psychiatric Sciences, 26(2), 129–138. doi:10.1017/S2045796016000172 27040595 PMC6998767

[r48] Morina, N., Akhtar, A., Barth, J., & Schnyder, U. (2018). Psychiatric disorders in refugees and internally displaced persons after forced displacement: A systematic review. Frontiers in Psychiatry, 9, 433. 10.3389/fpsyt.2018.00433 30298022 PMC6160546

[r49] Morris, S. B. (2023) ‘Meta-analysis in organizational research: A guide to methodological options. *Annual Review of Organizational Psychology and Organizational Behavior*, 10(1), 225–259. doi:10.1146/annurev-orgpsych-031921-021922.

[r50] Myung, S. K. (2023). How to review and assess a systematic review and meta-analysis article: A methodological study (secondary publication). Journal of Educational Evaluation for Health Professions, 20, 24. 10.3352/jeehp.2023.20.24 37619974 PMC10449599

[r51] National Institute of Mental Health. (2021, February 13). Integrating mental health care into health care systems in low- and middle-income countries and other low-resource settings. National Institutes of Health. https://www.nimh.nih.gov/funding/grant-writing-and-application-process/concept-clearances/2021/integrating-mental-health-care-into-health-care-systems-in-low-and-middle-income-countries-and-other-low-resources-settings?utm_source=chatgpt.com

[r52] Nijhuis, B. J., Reinders-Messelink, H. A., de Blécourt, A. C., Olijve, W. G., Groothoff, J. W., Nakken, H., & Postema, K. (2007). A review of salient elements defining team collaboration in paediatric rehabilitation. Clinical Rehabilitation, 21(3), 195–211. doi:10.1177/0269215506070674 17329277

[r53] Nordbrandt, M. S., Sonne, C., Mortensen, E. L., & Carlsson, J. (2020). Trauma-affected refugees treated with basic body awareness therapy or mixed physical activity as augmentation to treatment as usual-A pragmatic randomised controlled trial. PloS One, 15(3), e0230300. doi:10.1371/journal.pone.0230300 32163509 PMC7067472

[r54] Nosè, M., Ballette, F., Bighelli, I., Turrini, G., Purgato, M., Tol, W., … Barbui, C. (2017). Psychosocial interventions for post-traumatic stress disorder in refugees and asylum seekers resettled in high-income countries: Systematic review and meta-analysis. PLoS One, 12, e0171030. doi:10.5061/DRYAD.64KV7 28151992 PMC5289495

[r55] Oborn, E., & Dawson, S. (2010). Knowledge and practice in multidisciplinary teams: Struggle, accommodation and privilege. Human Relations, 63(12), 1835–1857. doi:10.1177/0018726710371237

[r56] Palic, S., & Elklit, A. (2011). Psychosocial treatment of posttraumatic stress disorder in adult refugees: A systematic review of prospective treatment outcome studies and a critique. Journal of Affective Disorders, 131(1–3), 8–23. doi:10.1016/j.jad.2010.07.005 20708804

[r57] Palic, S., & Elklit, A. (2009) An explorative outcome study of CBT-based multidisciplinary treatment in a diverse group of refugees from a Danish treatment centre for rehabilitation of traumatized refugees. Torture, 19(3), 248–70. 20065543

[r58] Perkins, D. D., & Schensul, J. J. (2017). Interdisciplinary contributions to community psychology and transdisciplinary promise. In M. A. Bond, I. Serrano-García, C. B. Keys, & M. Shinn (Eds.), APA handbook of community psychology: Theoretical foundations, core concepts, and emerging challenges (pp. 189–209). American Psychological Association. doi:10.1037/14953-009

[r59] Peters, J. L., Sutton, A. J., Jones, D. R., Abrams, K. R., & Rushton, L. (2007). Performance of the trim and fill method in the presence of publication bias and between-study heterogeneity. Statistics in Medicine, 26(25), 4544–4562. doi:10.1002/sim.2889 17476644

[r60] Piwowarczyk, L. A., & Ona, F. (2019). BeWell: quality assurance health promotion pilot. International *Journal of Health Care Quality Assurance*, 32(2), 321–331. doi:10.1108/IJHCQA-08-2017-0152 31017063

[r61] Purgato, M., Richards, J., Prina, E., Kip, A., Del Piccolo, L., Michencigh, G., Rimondini, M., Rudi, D., Vitali, F., Carta, M. G., Morina, N., Schena, F., & Barbui, C. (2021). Efficacy of physical activity interventions on psychological outcomes in refugee, asylum seeker and migrant populations: A systematic review and meta-analysis. Psychology of Sport and Exercise, 54, 101901. doi:10.1016/j.psychsport.2021.101901

[r62] Rohlof, H. G., Knipscheer, J. W., & Kleber, R. J. (2014). Somatization in refugees: A review. Social Psychiatry and Psychiatric Epidemiology, 49(11), 1793–1804. doi:10.1007/s00127-014-0877-1 24816685

[r63] Rosendahl, J., Alldredge, C. T., Burlingame, G. M., & Strauss, B. (2021). Recent developments in group psychotherapy research. American Journal of Psychotherapy, 74(2), 52–59. doi:10.1176/appi.psychotherapy.20200031 33745284

[r64] Rosenfield P. L. (1992). The potential of transdisciplinary research for sustaining and extending linkages between the health and social sciences. Social Science & Medicine (1982), 35(11), 1343–1357. doi:10.1016/0277-9536(92)90038-r 1462174

[r65] Sell, K., Hommes, F., Fischer, F., & Arnold, L. (2022). Multi-, inter-, and transdisciplinarity within the public health workforce: A scoping review to assess definitions and applications of concepts. International Journal of Environmental Research and Public Health, 19(17), 10902. doi:10.3390/ijerph191710902 36078616 PMC9517885

[r66] Shaw, S. A., Ward, K. P., Pillai, V., & Hinton, D. E. (2019). A group mental health randomized controlled trial for female refugees in Malaysia. American Journal of Orthopsychiatry, 89(6), 665–674. doi:10.1037/ort0000346 30035560

[r67] Silove, D., Manicavasagar, V., Coello, M., & Aroche, J. (2005). PTSD, depression, and acculturation. Intervention, 3, 46–50.

[r68] Silove, D., Ventevogel, P., & Rees, S. (2017). The contemporary refugee crisis: An overview of mental health challenges. World Psychiatry: Official Journal of the World Psychiatric Association (WPA), 16(2), 130–139. doi:10.1002/wps.20438 28498581 PMC5428192

[r69] Slobodin, O., & de Jong, J. T. (2015). Mental health interventions for traumatized asylum seekers and refugees: What do we know about their efficacy?. The International Journal of Social Psychiatry, 61(1), 17–26. doi:10.1177/0020764014535752 24869847

[r70] Stade, K., Skammeritz, S., Hjortkjær, C., & Carlsson, J. (2015). “After all the traumas my body has been through, I feel good that it is still working.”–Basic body awareness therapy for traumatised refugees. Torture, 25(1), 33–50. doi:10.7146/torture.v25i1.109507 26021346

[r71] Steel, Z., Chey, T., Silove, D., Marnane, C., Bryant, R. A., & van Ommeren, M. (2009). Association of torture and other potentially traumatic events with mental health outcomes among populations exposed to mass conflict and displacement: A systematic review and meta-analysis. JAMA, 302(5), 537–549. doi:10.1001/jama.2009.1132 19654388

[r72] Tay, A. K., Riley, A., Islam, R., Welton-Mitchell, C., Duchesne, B., Waters, V., … Ventevogel, P. (2019). The culture, mental health and psychosocial wellbeing of Rohingya refugees: A systematic review. Epidemiology and Psychiatric Sciences, 28(5), 489–494. doi:10.1017/S2045796019000192 31006421 PMC6998923

[r73] The World Bank. (2022). *World Bank country and lending groups country classification.* https://datahelpdesk.worldbank.org/knowledgebase/articles/906519-world-bank-country-and-lending-groups

[r74] Thomas, J., Thirlaway, K., Bowes, N., & Meyers, R. (2020). Effects of combining physical activity with psychotherapy on mental health and well-being: A systematic review. Journal of Affective Disorders, 265, 475–485. 10.1016/j.jad.2020.01.070 32090775

[r75] Thompson, C. T., Vidgen, A., & Roberts, N. P. (2018). Psychological interventions for post-traumatic stress disorder in refugees and asylum seekers: A systematic review and meta-analysis. Clinical Psychology Review, 63, 66–79. doi:10.1016/j.cpr.2018.06.006 29936342

[r76] Tol, W. A., Komproe, I. H., Jordans, M. J. D., Thapa, S. B, Sharma, B., & De Jong, J. T. V. M. (2009). Brief multi-disciplinary treatment for torture survivors in Nepal: A naturalistic comparative study. International Journal of Social Psychiatry, 55 *(*1), 39–56. doi:10.1177/0020764008091525 19129325

[r77] Turrini, G., Purgato, M., Acarturk, C., Anttila, M., Au, T., Ballette, F., … Barbui, C. (2019). Efficacy and acceptability of psychosocial interventions in asylum seekers and refugees: Systematic review and metaanalysis. Epidemiology and Psychiatric Sciences, 28, 376–388. doi:10.1017/S2045796019000027 30739625 PMC6669989

[r78] United Nations High Commissioner for Refugees Australia, (UNCHR). (2023). *Mid-year trends.* https://www.unhcr.org/au/mid-year-trends

[r79] Uphoff, E., Robertson, L., Cabieses, B., Villalón, F. J., Purgato, M., Churchill, R., & Barbui, C. (2020). An overview of systematic reviews on mental health promotion, prevention, and treatment of common mental disorders for refugees, asylum seekers, and internally displaced persons. The *Cochrane Database of Systematic Reviews*, 9(9), CD013458. doi:10.1002/14651858.CD013458.pub2 32885850 PMC8572368

[r80] Valentijn, P. P., Schepman, S. M., Opheij, W., & Bruijnzeels, M. A. (2013). Understanding integrated care: A comprehensive conceptual framework based on the integrative functions of primary care. International Journal of Integrated Care, 13, e010. doi:10.5334/ijic.886 23687482 PMC3653278

[r81] Van Wyk, S., & Schweitzer, R. D. (2013). A systematic review of naturalistic interventions in refugee populations. Journal of Immigrants and Minority Health. Advance online publication. doi:10.1007/s10903-013-9835-3 23666201

[r82] Verreault, K. (2017). Dance/Movement therapy and resilience building with female asylum seekers and refugees. Intervention (Amstelveen, Netherlands), 15(2), 120–135. doi:10.1097/WTF.0000000000000150

[r83] Stein, K. V., & Rieder, A. (2009). Integrated care at the crossroads-defining the way forward. International Journal of Integrated Care, 9, e10. doi:10.5334/ijic.315 19513179 PMC2691940

[r84] Wang, S.J., Bytyçi, A., Izeti, S., Kallaba, M., Rushiti, F., Montgomery, E., & Modvig, J. (2017). A novel bio-psycho-social approach for rehabilitation of traumatized victims of torture and war in the post-conflict context: A pilot randomized controlled trial in Kosovo. Conflict and Health, 10(1), 1–17. doi:10.1186/s13031-016-0100-y PMC5297130 28191034

[r85] Waring, J., Marshall, F., & Bishop, S. (2015). Understanding the occupational and organizational boundaries to safe hospital discharge. Journal of Health Services Research & Policy, 20(1 Suppl), 35–44. doi:10.1177/1355819614552512 25472988

[r86] White, C. C., Solid, C. A., Hodges, J. S., Boehm, D. H. (2015) Does integrated care affect healthcare utilization in multi-problem refugees. Journal of Immigrant Minority Health, 17, 1444–1450. doi:10.1007/s10903-014-0088-6 25150558

[r87] York, J., Rainforth, B., & Giangreco, M. F. (1990) Transdisciplinary teamwork and integrated therapy: Clarifying the misconceptions. Paediatric Physical Therapy, 2, 73–79. doi:10.1097/00001577-199002020-00003

