# Supplementary material for: Efficacy of integrated physical and psychological interventions on PTSD among forcibly displaced persons: a systematic review and meta-analysis
Source: Psychol Med. 2025 Apr 7;55:e109. doi: 10.1017/S0033291725000698 (PMC12094666; doi:10.1017/S0033291725000698)
Supplement: Chaudhari et al. supplementary material [file S0033291725000698sup001.docx]

Table of Contents

[Supplementary Material 1: Definitions](#_Toc169270724)

[Supplementary Material 2: Eligibility criteria for study inclusion](#_Toc169270725)

[Supplementary Material 3: Search Strategy](#_Toc169270726)

[Supplementary Material 4: List of excluded studies with reasons for exclusion](#_Toc169270727)

[Supplementary Material 5: Intervention Characteristics](#_Toc169270728)

[Supplementary Material 6: Risk of Bias](#_Toc169270729)

[Supplementary Material 7: Cumulative analysis](#_Toc169270730)

[Supplementary Material 8: Publication Bias](#_Toc169270731)

[Supplementary Material 9: Subgroup analysis](#_Toc169270732)

# Supplementary Material 1: Definitions

To define categories of cross-disciplinary integration, we reviewed literature and drew heavily from reviews by Rosenfield (1992), Choi & Pak (2006), Khalil (2021), and Sell (2022). From these key papers, two authors identified key indicators (see below), to classify the types of cross-disciplinary interaction and then categorised studies based on these indicators. Where there were disagreement authors came to a consensus following discussion.

|  | Definition | **Discipline boundary** | **Team communication/ interaction** | **Clinical decision making** | **Provider roles** |
| --- | --- | --- | --- | --- | --- |
| Multidisciplinary | Working in parallel or sequentially from disciplinary-specific frames to address common problems. Draws on knowledge from different disciplines but stays within the boundaries of those fields (Additive) | Maintained | None or limited communication between clinicians | Separate | Separate |
| Interdisciplinary | Working together but from individual disciplinary perspectives to address a common problem. Analyses, synthesized and harmonizes links between disciplines into a coordinated and coherent whole (Interactive) | Blurred | Regular communication between disciplines working together as a team at the point of delivery | Collaborative | Inter-related |
| Transdisciplinary | Working jointly using a shared conceptual framework that draws together concepts, theories, and approaches from multiple disciplines to create new knowledge and news solutions. Transcends traditional boundaries of each discipline (Holistic) | Dissolved | Constant communication between disciplines working together as a team in the design and delivery of the intervention | Shared /  Consensus | Inter-changeable  (role expansion, task shifting) |

# Supplementary Material 2: Eligibility criteria for study inclusion

|  | **Inclusion criteria** | **Exclusion criteria** |
| --- | --- | --- |
| Population | (i) refugees or asylum seekers  (ii) internally displaced persons, or  (iii) persons with who have experienced trauma as a result of living in conflict-affected regions. Older adolescents (14-19 years) and adults (≥18 years). No restriction on gender, ethnicity, or religion of participants. | Voluntary migrants; Economic migrants, or migrants who have migrated due to poverty, famine nor natural disasters; Children; Institutionalised populations (e.g., Inpatients) |
| Intervention | (i) Interventions with some combination of physical and psychological therapeutic modalities delivered concurrently, serially or as one single intervention. ‘Physical modalities’ are directed toward the treatment of physical disorders in the body and are applied through the physical body e.g. physiotherapy, exercise, physical activity. ‘Psychological modalities’ are directed toward treatment of mental or emotional disorders, and are applied through psychological means, such as counselling or psychotherapeutic programs and techniques (e.g., Integrative Adapt Therapy or Cognitive Behavioural Therapy) |  |
| Comparator | Wait list, treatment as usual, pharmaceutical care | No restriction |
| Outcomes | Quantitative outcomes of PTSD*. Studies were only included if these were measured by clinical diagnosis or using quantifiable, validated or commonly used rating scales. | Economic evaluations, process evaluation outcomes, qualitative outcomes |
| Study design | Randomised control trials (RCTs), cluster randomised control trials (cRCT), non-randomised controlled trials (NRCTs) | Pre-post intervention studies**; Qualitative Observational; cross-sectional; case-control, systematic reviews, meta-analyses |
| Language | English | Non-English publications |
| Publication date | 2000 to 01 Dec 2023*** | Before 2000 |
| Publication type | Research article reporting findings of eligible study designs | Conference abstracts, protocols, commentaries, unpublished literature, retracted articles |

*Quantitative outcomes of common mental disorders, stress or trauma related disorders, somatic disorders, and indicators of impaired function or wellbeing were included in the initial search, however only studies with quantitative outcomes for PTSD were presented in this systematic review and meta-analysis. This is depicted in the PRISMA diagram (see Methods)
** Pre-post intervention studies were included in the initial search and depicted in the PRISMA diagram. See Methods.
***Date when last searches were conducted.

# Supplementary Material 3: Search Strategy

| EMBASE |
| --- |
| 1. exp refugee/ |
| 1. exp asylum seeker/ |
| 1. refugee*.ti,ab,kw |
| 1. asylum.ti,ab,kw. |
| 1. migrant/ or emigrant/ or immigrant/ |
| 1. *migration/ or *immigration/ |
| 1. (force? adj2 (migrat* or immigrat* or emigrat*)).ti,ab,kw. |
| 1. (displac* adj1 (internal* or forced or mass or person* or people* or population*)).ti,ab,kw. |
| 1. floating population.ti,ab,kw |
| 1. ((post or after) adj migrat*).ti,ab,kw. |
| 1. exp warfare/ |
| 1. exp war/ |
| 1. (genocide or armed conflict* or mass execution* or mass violence).ti,ab,kw |
| 1. ((war or warfare) adj5 (abuse* or crime* or rape* or surviv* or victim*)).ti,ab,kw. |
| 1. (postconflict* or post conflict*).ti,ab,kw. |
| 1. exp torture/ |
| 1. (torture* or (politic* adj2 (detention or detainee? or persecut* or prison* or imprison* or violen*))).ti,ab,kw. |
| 1. exp human rights/ |
| 1. (humanitarian adj3 (aid or affair* or agenc* or assistance or catastrophe* or crisis or crises or disaster* or effort* or emergenc* or evacuation* or integration or reintegration or mission or organization* or organisation* or program* or relief or setting* or support* or task force or work*)).ti,ab,kw |
| 1. exp disaster/ |
| 1. 1 or 2 or 3 or 4 or 5 or 6 or 7 or 8 or 9 or 10 or 11 or 12 or 13 or 14 or 15 or 16 or 17 or 18 or 19 or 20 = 437 217 |
| 1. *mental disease/ |
| 1. Exp mental health/ |
| 1. Exp community mental health/ |
| 1. (mental* or psychiatr*).ti,kw. |
| 1. (mental* adj (health* or ill* or well* or disease* or disorder*)).ti,ab,kw. |
| 1. exp psychosocial disorder/ |
| 1. (psychological or psychosocial).ti,ab,kw. |
| 1. exp mood disorder/ |
| 1. mood disorder.ti,ab,kw |
| 1. ("depressive symptom*" or "affective disorder" or "affective symptom*" or dysthymi* or mood? or anxiety or agoraphobi* or panic or phobi*).ti,ab,kw. |
| 1. depression.ti,ab,kw |
| 1. exp grief/ |
| 1. (grief or grieving).mp |
| 1. exp anxiety disorder/ |
| 1. (anxiety disorder or phobia or panic disorder or panic attack or acute stress disorder or adjustment disorder or affective disorder or hypochondri*).ti,ab,kw. |
| 1. exp posttraumatic stress disorder/ |
| 1. post traumatic stress disorder.ti,ab,kw |
| 1. (PTSD or ((posttrauma* or post-trauma* or post trauma*) adj3 (stress* or disorder? or psych* or symptom*)) or acute* stress* or traumatic* stress* or stress disorder? or combat disorder? or war neuros*).ti,ab,kw |
| 1. (psycho* adj (stress* or distress*)).ti,ab,kw. |
| 1. (emotional adj (adjustment* or disorder*)).ti,ab,kw. |
| 1. exp substance abuse/ |
| 1. exp addiction/ |
| 1. exp somatoform disorder/ |
| 1. 21 or 22 or 23 or 24 or 25 or 26 or 27 or 28 or 29 or 30 or 31 or 32 or 33 or 34 or 35 or 36 or 37 or 38 or 39 or 40 or 41 or 42 or 43 |
| 1. (Holistic or integrated or combined).ti,ab,kw |
| 1. exp psychotherapy/ |
| 1. Psychotherapy.ti,ab,kw. |
| 1. exp physiotherapy/ |
| 1. exp exercise/ |
| 1. stretching.ti,ab,kw. |
| 1. progressive muscle relaxation.ti,ab,kw. |
| 1. dance movement therapy.ti,ab,kw. |
| 1. yoga.ti,ab,kw. |
| 1. kinesio*.ti,ab,kw. |
| 1. movement therap*.ti,ab,kw. |
| 1. mitchell method.ti,ab,kw. |
| 1. bioenergetic exercise.ti,ab,kw. |
| 1. body-orientated psych*.ti,ab,kw. |
| 1. body-based psych*.ti,ab,kw. |
| 1. movement-based psych*.ti,ab,kw. |
| 1. mind-body.ti,ab,kw. |
| 1. (moto*senso* or senso*moto).ti,ab,kw. |
| 1. somato*senso*.ti,ab,kw. |
| 1. somatic experiencing.ti,ab,kw. |
| 1. somatic transformation.ti,ab,kw. |
| 1. movement awareness.ti,ab,kw. |
| 1. body consciousness.ti,ab,kw. |
| 1. body awareness.ti,ab,kw. |
| 1. neuroemotional technique.ti,ab,kw. |
| 1. (autogenic and (training or retraining)).ti,ab,kw. |
| 1. perceptual training.ti,ab,kw. |
| 1. (breath*work or breathing exercise*).ti,ab,kw. |
| 1. (47 or 48) AND (49 or 50 or 5) |
| 1. 52 or 53 or 54 or 55 or 56 or 57 or 58 or 59 or 60 or 61 or 62 or 63 or 64 or 65 or 66 or 67 or 68 or 69 or 70 or 71 or 72 or 73 |
| 1. 46 or 74 or 75 = 1 590 472 |
| 1. 21 and 45 and 76 |

# Supplementary Material 4: List of excluded studies with reasons for exclusion

| First Author, Year | Reason for exclusion |
| --- | --- |
| Abdollahi 2017 | Ineligible participants |
| Ahmed 2019 | Ineligible study design |
| Akhtar 2021 | Ineligible intervention |
| Altawil 2018 | Ineligible intervention |
| Andersen 2017 | Ineligible participants |
| Andersen 2020 | Ineligible intervention |
| Ansbro 2021 | No full text |
| Arcel 2003 | Ineligible study design |
| Askovic 2019 | Ineligible intervention |
| Barron 2021 | Ineligible intervention |
| Benz 2019 | Ineligible participants |
| Blaauwendraat 2017 | Ineligible participants |
| Bleile 2021 | Ineligible study design; Ineligible participants; Ineligible outcome |
| Bolton 2007 | Ineligible intervention |
| BroddaJansen 2011 | No full text |
| Bruno 2019 | Ineligible intervention; Ineligible study design; Ineligible outcome |
| Burchert 2019 | Ineligible intervention; Ineligible study design; Ineligible outcome |
| Callaghan 1993 | Ineligible study design |
| Cardeli 2020 | Ineligible intervention |
| Carlsson 2005 | Another publication part of same study included |
| Chemali 2017 | Ineligible participants |
| Chowdhary 2007 | Ineligible intervention |
| Classen 2021 | Ineligible participants |
| Connolly 2011 | Ineligible intervention |
| Copolov, & Knowles (2021) | Ineligible intervention |
| Corna 2019 | Ineligible intervention |
| Cornelio-Flores 2018 | Ineligible participants |
| Dalgaard 2020 | Ineligible intervention; Ineligible study design; Ineligible outcome |
| Diab 2017 | Ineligible study design |
| Dibaj 2017 | Ineligible Study design |
| Doumit 2020 | Ineligible intervention |
| Drozdek 2010 | Another publication part of same study included |
| Drozdek 2012 | Another publication part of same study included |
| Dyer 2019 | Ineligible intervention |
| El Khodary 2020 | Ineligible participants |
| Emminghaus 2016 | Ineligible study design |
| Fine 2021 | Ineligible intervention |
| Folkes 2002 | Ineligible intervention |
| Gagnon 2014 | Ineligible participants |
| Gamble 2020 | Ineligible participants |
| Gerbarg 2011 | Ineligible study design |
| Golchert 2019 | Ineligible intervention |
| Grasser 2018 | Ineligible study design |
| Grasser 2019 | Insufficient experimental data; Ineligible participants |
| Griggs 2022 | Ineligible intervention |
| Grodin 2008 | Ineligible study design |
| Hammad 2020 | Ineligible intervention |
| Han 2012 | Ineligible intervention |
| Hancock, 2009 | Ineligible intervention |
| Hardi 2011 | Ineligible intervention; Ineligible study design |
| Harris 2007 | Ineligible study design |
| Hasanovic 2012 | Ineligible intervention |
| Highfield 2012 | Ineligible intervention; Ineligible study design |
| Hill 2019 | Ineligible intervention |
| Horrigan 2008 | Ineligible intervention; Ineligible study design |
| Humayun 2017 | Ineligible intervention; Ineligible outcome |
| Husby 2020 | Ineligible intervention |
| Jewell 2013 | Ineligible participants |
| Jindani 2015 | Ineligible participants |
| Jordan 2010 | Ineligible participants |
| Jordan 2013 | Ineligible participants |
| Jordans 2014 | Ineligible study design |
| Jordans, Komproe, Tol, Kohrt, Luitel, Macy & de Jong (2010) | Ineligible participants |
| Jordans, Tol, Susanty, Ntamatumba, Luitel, Komproe & de Jong (2013) | Ineligible participants |
| Kananian 2020 | Ineligible intervention |
| Kellner 2012 | Ineligible participants |
| Keough 2004 | Ineligible intervention; Ineligible participants |
| Keshk 2021 | Ineligible intervention |
| Khamis 2004 | Ineligible participants |
| Kieft 2008 | Ineligible intervention; Ineligible outcome |
| Knappe 2019 | Ineligible intervention |
| Koch 2009 | Ineligible study design |
| Kotsioni 2017 | No full text |
| Layne 2008 | Ineligible intervention |
| Leiler 2020 | Ineligible intervention |
| Lewis 2007 | Ineligible study design |
| Liedl 2011 | Retracted article |
| Loughry 2006 | Ineligible intervention |
| Madsen 2016 | Ineligible study design |
| Maffia 2008 | Ineligible intervention; Ineligible study design |
| Mahmooth 2018 | Ineligible intervention |
| McBeth 2014 | Ineligible participants |
| McBeth 2012 | Ineligible participants |
| Merom 2008 | Ineligible participants |
| Miller 2020 | Ineligible intervention |
| Milosevic 2012 | Ineligible study design |
| Mitchell 2014 | Ineligible participants |
| Mitchels 2003 | Ineligible study design |
| Morina 2012 | Retracted article |
| Mufson 2008 | Ineligible intervention |
| Mughal 2015 | Ineligible intervention |
| Muller 2009 | Retracted article |
| NCT03515564 2018 | Ineligible participants |
| NCT04244864 2020 | Ineligible intervention |
| Negron 2018 | Ineligible intervention |
| Nordin & Perrin (2019) | Ineligible study design |
| O'Callaghan 2015 | Ineligible intervention |
| O'Connell 2012 | Ineligible intervention; Ineligible study design |
| Pentecost 2015 | Ineligible participants |
| Philipps 2019 | Ineligible intervention; Ineligible study design; Ineligible outcome |
| Portokaloglou 2018 | Ineligible study design |
| Powell 2021 | Ineligible outcome |
| Purgato 2017 | Ineligible study design |
| Quinlan 2016 | Ineligible intervention |
| Quosh 2013 | Ineligible intervention |
| Quosh 2013 | Ineligible study design |
| Rahapsari 2019 | Ineligible intervention |
| Rahman 2019 | Ineligible intervention |
| Ramya 2011 | Ineligible participants |
| Rhodes 2016 | Ineligible participants |
| Richards 2014 | Ineligible intervention |
| Romao 2021 | Ineligible intervention; Ineligible study design |
| Rose 2011 | Ineligible intervention; Ineligible study design |
| Russell 2021 | Ineligible intervention; Ineligible outcome |
| Shaw 2021 | Ineligible intervention |
| Shaw Abstract 2023 | Ineligible intervention |
| Shultz 2019 | Ineligible intervention |
| Sleptsova, Woessmer, Grossman, & Langewitz (2013) | Ineligible participants |
| Smeets 2008 | Ineligible participants |
| Smeets, Vlaeyen, Hidding, Kester, van der Heijden & Knottnerus (2008) | Ineligible participants |
| Stammel 2017 | Ineligible intervention |
| Staples 2008 | Ineligible participants |
| Stein et al 2003 | Ineligible participants |
| Stepakoff 2006 | Ineligible intervention; Ineligible study design |
| Stewart 2015 | Ineligible intervention; Ineligible outcome |
| Sullivan 2019 | Ineligible outcome |
| Sunallah, van den Boogaard, Lakis, Rinchey & Saavedra (2021) | Ineligible intervention; Ineligible study design |
| Taloyan 2013 | Ineligible participants |
| Tarannum, Elshazly, Harlass & Ventevogel (2019) | Ineligible intervention |
| Tay 2020 | Ineligible intervention |
| Tol 2017 | Ineligible intervention; Ineligible participants |
| Tol, Komproe, Jordans, Vallipuram, Sipsma, Sivayoka, Macy & de Jong (2013) | Ineligible participants |
| Tol, Komproe, Jordans, Ndayisaba, Ntamatumba, Sipsma, Smallgange, Macy & de Jong (2014) | Ineligible participants |
| Tol, Komproe, Susanty, Jordans, Macy, & De Jong (2010) | Ineligible participants |
| Van der Waerden, Hoefnagels, Hosman, Souren & Jansen (2013) | Ineligible participants |
| VanDerKolk 2014 | Ineligible participants |
| Vasudev 2020 | Ineligible participants |
| Wei-LunCHANG 2018 | Ineligible Outcomes |
| Weine, Ware, Hakizimana, Tugenberg, Currie, Dahnweih, Wagner, Polutnik & Wulu (2014) | Ineligible intervention; Ineligible study design |
| Womersley 2019 | Ineligible intervention |

## Studies excluded from Systematic Review for PTSD outcome

| **First Author, Year** | **Reason for exclusion** |
| --- | --- |
| Phaneth 2014 | No PTSD outcome |
| Salihu 2021 | No PTSD outcome |
| Danneskiold-Samsoe 2007 | No PTSD outcome |
| Gueron 2021 | No PTSD outcome |
| Poudel-Tandukar 2021 | No PTSD outcome |
| Poudel-Tandukar 2021 | No PTSD outcome |

# Supplementary Material 5: Intervention Characteristics

| Author, date | Intervention | Intervention Components | | | | Integration type |
| --- | --- | --- | --- | --- | --- | --- |
|  |  | **Psychological** | **Physical** | **Combined** | **Other** |  |
| Aizik-Reebs et al., 2021 | Mindfulness-Based Trauma Recovery  for Refugees (MBTR-R) |  |  | Formal and informal mindfulness practices (e.g., body scan, sitting meditation, mindful movement, 3-min breathing space), although with key trauma-sensitive adaptations, experiential inquiry-based discussions of all in-session practices, and home practice via web-based audio recordings and handouts |  | Transdisciplinary |
| Carlsson et al., 2010 |  | Psychotherapy, social counselling | Physiotherapy |  | Medical help | Multidisciplinary |
| Drožđek et al., 2012 | Den Bosch Group treatment - psychodynamic, cognitive-behavioural, and  supportive approaches | Psychotherapy |  | Psychomotor body therapy (dance movement therapy) | Art therapy, and music therapy.  Psychotropic medication were administered in the majority (95%) of participants, both in the treatment and the waiting list conditions. | Interdisciplinary |
| Eskici et al., 2023 | Culturally Adapted Cognitive Behavioral Therapy (CA-CBT) | CBT + Psychoeducation |  | Muscle relaxation with visualisation, emotion regulation techniques, mindfulness, imagery, abdominal breathing and smiling exercise (facial expression mindfulness); smiling and belly laughing practices; walking meditation; emotion regulation techniques such as mindfulness, stretching, and adaptive imagery. |  | Transdisciplinary |
| Gordon et al., 2004 | Mind-Body Skills Group (MBSG) |  |  | Meditation, biofeedback, drawings, autogenic training, guided imagery, genograms, movement, and breathing techniques |  | Transdisciplinary |
| Gordon et al., 2008 | Mind-Body Skills Group (MBSG) |  |  | Guided imagery, relaxation techniques, several forms of meditation, autogenic training, biofeedback and active techniques (fast, deep breathing; shaking; and dancing), written expression and genograms. |  | Transdisciplinary |
| Gordon et al., 2016 |  |  |  | Meditation, guided imagery, breathing techniques,  autogenic training, biofeedback, genograms, and Self-expression through words, drawings, and movement (shaking, dancing) |  | Transdisciplinary |
| Harlacher et al., 2016 | Pain school | Psychoeducation | Psychoeducation | Psychoeducation |  | Interdisciplinary |
| Hasha et al., 2020 | Physiotherapy Activity and Awareness Intervention (PAAI) |  | General physiotherapy and exercises including coordination exercises (components of PAAI including) | Mindfulness exercises, Relaxation, and breathing exercises (components of PAAI including) |  | Transdisciplinary |
| Kananian et al., 2020 | Culturally Adapted Cognitive Behavioral Therapy Plus Problem Management (CA-CBT+) | CBT + Problem management |  | Yoga and stretching exercises, meditation techniques including mindfulness breathing and loving–kindness meditation as well as culturally appropriate guided imagery (e.g., “Persian garden”). |  | Transdisciplinary |
| Nordbrandt et al., 2020 | Multidisciplinary program | Psychotherapy (CBT with elements of acceptance and commitment therapy, stress management and mindfulness) | Intervention B) Basic Body Awareness Training: slow, guided movements aiming at normalising and improving balance, muscle tension, free breathing and awareness  Intervention A) Mixed physical activity (basic exercises focusing on improving strength, endurance, balance and coordination) | Breathing and body awareness exercises and education on body awareness in coping with pain and stress (in BBAT) | All groups received: 10 sessions with a medical doctor - Pharmacological treatment if needed and providing psychoeducation on a wide range of topics such as explaining symptoms of PTSD and depression; advice on how to improve sleep. | Multidisciplinary |
| Palic et al., 2009 |  | Psychotherapy (CBT with focus on exposure and some elements of EDMR) | Physiotherapy (BBAT) | Breathing and body awareness exercises and education on body awareness in coping with pain and stress (in BBAT) | Participants also were receiving care from municipality GP (pharmacotherapy) and social workers | Multidisciplinary |
| Shaw et al., 2019 | Somatic-focused culturally adapted cognitive-behavioural therapy (CBT) |  |  | Skills related to the following main topics: emotional regulation, stretching and relaxation, breathing, mindfulness, visualization, and managing anger and worry. |  | Transdisciplinary |
| Stade et al., 2015 |  |  | Physiotherapy (BBAT) | Breathing and body awareness exercises and education on body awareness in coping with pain and stress (in BBAT) |  | Transdisciplinary |
| Tol et al., 2009 | Multidisciplinary program | Psychosocial counselling, psychoeducation | Physiotherapy | Relaxation exercises, yoga, techniques based on energy psychology (Emotional Freedom Technique) | Techniques based on energy psychology (Emotional Freedom Technique) | Multidisciplinary |
| Wang et al., 2016 | Multidisciplinary program | Psychotherapy (CBT based on an adapted prolonged exposure therapy manual) and including breathing exercise with an emWave biofeedback device | Physiotherapy |  | Both groups were given a vitamin | Multidisciplinary |

# Supplementary Material 6: Risk of Bias

| RCT  (ROB v2) | Domain 1. Randomization process | Domain 2. Deviations from intended interventions | Domain 3. Missing outcome data | Domain 4. Measurement of the outcome | Domain 5. Selection of the reported result | **Domain 6. Overall bias (Algorithm)** |
| --- | --- | --- | --- | --- | --- | --- |
| Gordon et al., 2008 | LOW | SOME | LOW | SOME | SOME | HIGH |
| Harlacher et al., 2016 | SOME | LOW | LOW | SOME | SOME | HIGH |
| Hasha et al., 2020 | LOW | LOW | SOME | SOME | LOW | SOME |
| Nordbrandt et al., 2020 | LOW | LOW | SOME | SOME | LOW | SOME |
| Shaw et al., 2019 | LOW | LOW | LOW | SOME | LOW | SOME |
| Wang et al., 2016 | LOW | LOW | LOW | LOW | LOW | LOW |
| Kananian et al., 2020 | LOW | LOW | LOW | SOME | LOW | LOW |
| Aizik-Reebs et al., 2021 | LOW | LOW | LOW | SOME | LOW | LOW |
| Eskici et al., 2023 | LOW | LOW | LOW | SOME | SOME | SOME |
| Drozdek et al., 2012 | SOME | LOW | LOW | SOME | LOW | SOME |
| Tol et al., 2009 | SOME | LOW | LOW | SOME | LOW | SOME |

| nRCT  (EPHPP) | REVIEWER | SELECTION BIAS | STUDY DESIGN | CONFOUNDERS | BLINDING | DATA COLLECTION METHODS | WITHDRAWALS AND DROP-OUTS | INTERVENTION INTEGRITY | ANALYSES | **GLOBAL RATING** |
| --- | --- | --- | --- | --- | --- | --- | --- | --- | --- | --- |
| Carlsson et al., 2010 | STRONG | MOD | STRONG | WEAK | STRONG | WEAK | WEAK | WEAK | WEAK | MOD |
| Stade et al., 2015 | WEAK | MOD | STRONG | WEAK | STRONG | MOD | MOD | WEAK | WEAK | MOD |
| Palic et al., 2009 | MOD | MOD | STRONG | WEAK | MOD | STRONG | WEAK | MOD | MOD | STRONG |
| Gordon et al., 2004 | WEAK | MOD | STRONG | WEAK | STRONG | MOD | MOD | MOD | WEAK | MOD |
| Gordon et al., 2016 | MOD | MOD | STRONG | WEAK | STRONG | WEAK | MOD | STRONG | WEAK | MOD |

# Supplementary Material 7: Publication Bias

## Figure 7.1: Trim and fill plot

# Supplementary Material 8: Subgroup analysis

## Figure 8.1: Forrest Plot - Subgroup analysis by PTSD Measure (HTQ vs. Other)

## Figure 8.2: Forrest Plot - Subgroup analysis by type of integration (Multidisciplinary vs. Interdisciplinary vs. Transdisciplinary)

## Figure 8.3: Forrest Plot - Subgroup analysis by service delivery (Group vs. Individual)

## Figure 8.4: Forrest Plot - Subgroup analysis by service delivery (Type of personnel: Health professional vs. Other)
